# Investigation of Crystallization and Relaxation Effects in Coarse-Grained Polyethylene Systems after Uniaxial Stretching

**DOI:** 10.3390/polym13244466

**Published:** 2021-12-20

**Authors:** Dirk Grommes, Martin R. Schenk, Olaf Bruch, Dirk Reith

**Affiliations:** 1 Institute of Technology, Resource and Energy-Efficient Engineering (TREE), Bonn-Rhein-Sieg University of Applied Sciences, Grantham-Allee 20, 53757 Sankt Augustin, Germany; dirk.grommes@h-brs.de (D.G.); martin.schenk@h-brs.de (M.R.S.); olaf.bruch@h-brs.de (O.B.); 2 Dr. Reinold Hagen Stiftung, Kautexstrasse 53, 53229 Bonn, Germany; 3 Fraunhofer Institute for Algorithms and Scientific Computing (SCAI), Schloss Birlinghoven, 53754 Sankt Augustin, Germany

**Keywords:** mesoscale coarse-graining, polyethylene, uniaxial stretching, relaxation, shrinkage, crystallization, local chain orientation

## Abstract

In this study, we investigate the thermo-mechanical relaxation and crystallization behavior of polyethylene using mesoscale molecular dynamics simulations. Our models specifically mimic constraints that occur in real-life polymer processing: After strong uniaxial stretching of the melt, we quench and release the polymer chains at different loading conditions. These conditions allow for free or hindered shrinkage, respectively. We present the shrinkage and swelling behavior as well as the crystallization kinetics over up to 600 ns simulation time. We are able to precisely evaluate how the interplay of chain length, temperature, local entanglements and orientation of chain segments influences crystallization and relaxation behavior. From our models, we determine the temperature dependent crystallization rate of polyethylene, including crystallization onset temperature.

## 1. Introduction

Polymers are of great importance in industrial and consumer related applications. Their low costs, easy processability, and good performance characteristics give polymers versatile usage options. Yet individual component design is a difficult procedure and in many cases is characterized by strong compromises due to their distinctive time, temperature, and load-dependent behavior. For the development of an optimized product at minimum material usage, the use of computer-aided-engineering (CAE) has become increasingly important over the past several years. Simulation-based product tests using the method of finite elements (FE) are already state of the art. Several properties of the final part such as the deformation behavior are predictable. Nevertheless, simulation results strongly depend on the input data for the analysis procedure [1,2,3,4,5]. Clearly the material description, consisting of the choice of the material model and the related material properties, influences the results. In many cases, specific material properties, which are generally determined by experiments, are not available. Furthermore, performing the necessary experiments is associated with high costs, especially if different types of experiments are needed to fully characterize the material’s behavior [1,2,3].

An alternative way to overcome that limitation is the use of molecular dynamics (MD) simulation methods [6,7]. Modelling a specific polymer on the microscale enables the determination of material parameters needed for the simulations on the macroscopic scale.

One of the most crucial factors in real-life polymer processing is the characterization of the shrinkage behavior due to thermally and mechanically induced stress during processing. Theses stresses occur in every type of plastics processing [8]. For example, during the extrusion and inflation stages in the widespread extrusion blow molding process [9,10] or during sheet deformation in the thermoforming process [11]. In this work, we therefore investigate the shrinkage, relaxation and crystallization behavior of polymer systems under different temperature and loading conditions, which resemble real-life processing conditions. Concerning which material to investigate, the choice was made for polyethylene (PE) as it is widely spread in industrial and consumer application. Because of its simple structure, it can be easily modeled on the microscopic scale.

In contrast to previous studies, our approach is to discuss both the strain (stress) induced crystallization behavior as well as the resulting changes of the system size to give an estimate of the shrinkage behavior after prior loading. As we focus on mimicking macroscopic processing conditions we stretch, cool and relax our systems under two different approaches that both typically occur in real-life processing: (1) “free conditions”, where the system (the real part) is able to shrink immediately after stretching; (2) “fixed conditions”, where the box size (the part shape) is fixed in every direction of space to mimic the effect of a mold constraint. Only after cooling is finished (and the part is demolded), the box (the part) is allowed to change its shape and size.

In the past, some MD studies have been performed to investigate the relaxation behavior of polymers after prior loading. As for our systems, we expect that crystallization will play an important role, an overview about recent studies on that topic is also appropriate.

Hsu and Kremer [12,13] investigate the relaxation behavior of a generic polymer model after large uniaxial stretching. Their main focus is on the analysis of the primitive path and the stress relaxation behavior. Related studies of highly oriented polymers are found in [14,15,16].

There exist different studies that use united atom (UA) models of polyethylene for the investigation of crystallization in stretched systems [17,18,19,20]. These studies only focus on crystallization effects and leave relaxation effects aside.

Moyassari et al. [21,22] use a coarse-grained (CG) model, where they map two CH_2_ units in one super-bead. They investigate the crystallization behavior of different bimodal [21] and short-branched [22] polyethylene melts starting from the pure amorphous, quiescent state. They perform long simulation runs up to 800 ns. Verho et al. [23] also focus on the crystallization of polyethylene. By using a UA model, they start from a given crystal seed to initialize crystallization. Still, very long simulation runs up to 1200 ns are necessary to capture the relaxation behavior, at least in part. Hall et al. [24] investigate different properties of polyethylene by the use of a CG model (three CH_2_ units into one super-bead). Inter alia, they observe crystallization behavior from quiscient melt in long simulation runs.

An overview about different UA force fields and their influence on the simulation of crystallization is given by Hagita et al. [25]. By investigating eight common force fields, they show that crystallization effects in simulation models strongly depend on the choice of the force field.

## 2. Simulation Methodology

### 2.1. Force Field

The force field [26] that we use for the investigation of relaxation and crystallization effects was previously used for the simulation of tensile tests on the micro scale [27]. It was shown that it is suitable for the evaluation of orientation and entanglement effects that drive the mechanical behavior of polyethylene. Therefore it is an ideal starting point for the investigation of strongly stretched systems and their relaxation behavior.

The bonded interaction as well as Lennard Jones (LJ) parameters for CG polyethylene description are presented in Table 1. LJ parameters in [26] are optimized to have good agreement with experimental density and heat of vaporization. For more accuracy [26] defines the LJ parameters depending on the particle position (end or middle position in the chain). Therefore two types of CG beads (CG_mid_, CG3_end_) are defined. The first- and second-neighbour beads are excluded from the non-bonded interactions. Additionally, there is a third-neighbour LJ interaction with different parameters. The cut-off distance $r_{c}$ is taken as 2.5 times the value of σ of the middle bead. All non-bonded parameters are summarized in Table 1.

Special attention is paid to the proper equilibration of polymer melts as this is a highly non-trivial task. We apply the equilibration procedure from Moreira et al. [28] and Auhl et al. [29]. As shown in our previous work [27] we are able to adopt that procedure for the equilibration of our chemically specific force field. By verifying the static melt structure factor and the mean square internal distance of the final systems, we make sure that these are well equilibrated. The results of the equilibration are found in [27]. We equilibrate and further investigate four different system sizes with various number of chains *M* and chain length *N* (M×N: 1000×500, 100×1000, 500×1000, 250×2000). The investigated chain lengths cover a range where systems have distinctively different behavior. Shortest investigated chain length (N=500) is suitable to serve as an example for showing that these systems do not represent real-life polymer behavior under excessive stretching. On the contrary, the longest investigated chains (N=2000) give a much better estimate of polymer behavior with respect to experimental results. Our system size of 500,000 particles ensures reasonable computation times. To keep total system sizes constant, the number of chains per system is 1000 (for N=500), 500 (for N=1000) and 250 (for N=2000), respectively. We do not investigate longer chains as systems would then consist of less than 250 chains, which would lead to less reliable statistics. The only exception is the system with 100 chains at chain length 1000, which we use to make the long time scale (up to 600 ns) accessible within a reasonable time frame. Our equilibrated systems have density of the amorphous phase at 293 K ($\rho_{\mathrm{amorph},293K}$), melt density ($\rho_{493K}$), coefficient of thermal expansion (CTE) and glass transition temperature (*T*g) in good agreement with experimental results (Table 2).

### 2.2. Simulation Procedure

Molecular dynamics simulations are performed using the ESPResSo++ package [34,35]. Starting with the equilibrated systems our simulation procedure consists of three steps: (1) stretching of the samples of amorphous melt; (2) quenching of the samples to a specific temperature at two different conditions; (3) final relaxation of the samples.

In the first step, we continuously stretch the systems in the melt state at 500 K up to a degree of stretching λ=6 at an initial strain rate of $1\;\cdot \;10^{8}\;s^{-1}$. We therefore extended the ESPResSo++ source code in order to enable uniaxial stretching of the box under consideration of simultaneous free transversal contraction. The selected strain rate ensures strong orientations of chain segments without over-pronounced disentanglement of chains. The stretching is performed under usage of the Berendsen barostat for the transversal directions ($\tau_{\mathrm{baro}}=10\;\mathrm{ps}$), Berendsen thermostat ($\tau_{\mathrm{thermo}}=1\;\mathrm{ps}$) and periodic boundary conditions (rectangular box). Note that strong stretching of chains includes a potential risk of bond crossing. However, due to the still moderate level of stretching in our simulations (cf. [19,22]), we do not expect bond crossing to happen.

The cooling of the samples while releasing the tensile strain is done in two distinctively different ways which mimic different real-life processing conditions. The first option is choosing “free” boundary conditions, which allow the previously stretched melt to contract instantaneously after λ=6 is attained. This is done by using an anisotropic Berendsen barostat ($\tau_{\mathrm{baro}}=1000\;\mathrm{ps}$) and setting the pressure to 1 bar in every direction of space. Simultaneously, we cool the systems down to the target temperature (353.15 K, 293.15 K, 233.15 K) within a time frame of 10 ns by using the Berendsen thermostat ($\tau_{\mathrm{thermo}}=1\;\mathrm{ps}$). The second option for the cooling procedure is a “fixed” boundary condition: Instead of allowing the system to contract immediately after stretching, we here fix the box dimensions during cooling by not using a barostat. In the same way as for free conditions, the cooling time is set to 10 ns by use of the Berendsen thermostat at $\tau_{\mathrm{thermo}}=1\;\mathrm{ps}$. This allows for the internal relaxation of stress (due to conformational relaxation) while the system is not able to change its size and shape.

The final step of our simulation procedure is the observation of the system evolution after the cooling is finished. We therefore let each system evolve by using the anisotropic Berendsen barostat at the specific target temperature, set by the Berendsen thermostat. This means that the systems, which are cooled under fixed boundary conditions, are now able to change their shape and size. Integration time step for all simulation steps is set to 4 fs.

### 2.3. Evaluation of the Microscopic Structure

Evaluation of the microscopic structure of the polymer systems is essential for the interpretation of macroscopic effects. We evaluate the orientation behavior both on the local as well as on the global scale with respect to the stretching direction. We define the orientation factor δ according to Equation (1) as the projection of either the normalized bond vector ${\vec{r}}_{\mathrm{bond}}/\left|{\vec{r}}_{\mathrm{bond}}\right|$ or analogously the chain end-to-end vector ${\vec{r}}_{\mathrm{end}\text{-}\mathrm{to}\text{-}\mathrm{end}}/\left|{\vec{r}}_{\mathrm{end}\text{-}\mathrm{to}\text{-}\mathrm{end}}\right|$ on the unit vector ${\vec{e}}_{\mathrm{tensile}}$ in pulling direction. The brackets in Equation (1) indicate the average over all bond or chain end-to-end vectors, respectively. This definition is widely used for the evaluation of orientations of chain segments [17,18,19,21].

(1) $$\delta =\frac{3}{2}\langle {\left(\frac{\left|\vec{r}\;\cdot \;{\vec{e}}_{\mathrm{tensile}}\right|}{\left|\vec{r}\right|}\right)}^{2}\rangle -\frac{1}{2}$$

For the evaluation of entanglements we use the primitive path analysis (PPA) introduced by Everaers et al. [36], based on the assumptions of the tube model [37,38,39]. The PPA algorithm has become a standard tool in computer simulation. [40] For PPA, chain-ends are fixed in space and all interactions except for the bond and interchain excluded volume interactions are switched off. The harmonic bond interaction is replaced by a FENE (finite extensible non-linear elastic spring) interaction according to [34] with attractive force strength $K=1000\;\mathrm{kJ}\;{\mathrm{mol}}^{-1}{\mathrm{nm}}^{-2}$, displacement parameter $r_{0}=0\;\mathrm{nm}$ and size parameter $r_{\max}=0.55\;\mathrm{nm}$. The energy of the system is minimized so that, finally, chains shrink to straight segments with clear kinks. The length of the primitive path (PP) $\langle L_{\mathrm{pp}}\rangle =\left(N-1\right)\langle b_{\mathrm{pp}}\rangle$, where $\langle b_{\mathrm{pp}}\rangle$ is the PP bond length, represents the contour length of the tube. The tube radius $a_{\mathrm{pp}}$ is defined as $a_{\mathrm{pp}}=\langle R_{\mathrm{ee}}^{2}\rangle /\langle L_{\mathrm{pp}}\rangle$, where $\langle R_{\mathrm{ee}}^{2}\rangle$ is the mean squared end-to-end distance of the chains. From that the entanglement length $N_{e}$ according to [36] is:

(2) $$N_{e}=\frac{a_{\mathrm{pp}}}{\langle b_{\mathrm{pp}}\rangle }=\left(N-1\right)\frac{\langle R_{\mathrm{ee}}^{2}\rangle }{{\langle L_{\mathrm{pp}}\rangle }^{2}}$$

For our evaluations we define the number of entanglements *Z* per chain as the ratio $N/N_{e}$ (cf. [12]). This relation allows for an estimate of how entangled the systems with different chain lengths are. Related methods for the investigation of entanglements are presented and extensively discussed in [40,41,42].

Furthermore, we determine the crystallinity $x_{\mathrm{cryst}}$ of the systems from a microscopic and a macroscopic definition. The determination on the microscopic scale is done by the use of an own developed procedure. We sample over all beads and determine the degree of crystallinity as follows:

(1) The current bead *i* is closer to a neighboring bead *j* than $0.975\;\cdot \;2^{1/6}\sigma$. Bonded first neighboring beads are excluded here;

(2) The orientation factor $\delta_{\mathrm{cryst}}$ between vectors ${\vec{V}}_{i-1,i+1}$ and ${\vec{V}}_{j-1,j+1}$ (vectors between first neighboring beads of bead *i* and bead *j*) according to:

(3) $$\delta_{\mathrm{cryst}}=\frac{3}{2}{\left(\frac{\left|{\vec{V}}_{i-1,i+1}\;\cdot \;{\vec{V}}_{j-1,j+1}\right|}{\left|{\vec{V}}_{i-1,i+1}\right|\;\cdot \;\left|{\vec{V}}_{j-1,j+1}\right|}\right)}^{2}-\frac{1}{2}$$

is larger than 0.9;

(3) We define the microscopic crystal stem length $n_{\mathrm{stem}}$ as the number of consecutive beads within a chain that fulfil criterion (1) and (2). All beads that belong to stems with $n_{\mathrm{stem}}\geq 3$ are regarded as being in a crystalline state. Our investigations reveal that in purely amorphous systems the likelihood of finding structures with $n_{\mathrm{stem}}\geq 3$ is very low and rises quickly for $n_{\mathrm{stem}}<3$;

(4) By counting the number of crystalline beads $N_{\mathrm{cb}}$ according to (3) we determine the degree of crystallinity $x_{\mathrm{cryst},\mathrm{micro}}$ by dividing the number of crystalline beads $N_{\mathrm{cb}}$ by the number of total beads $N_{\mathrm{total}}$;

The macroscopic definition is calculated from the ratio of the total density ρ, the pure amorphous $\rho_{\mathrm{am}}$ and pure crystalline densities $\rho_{\mathrm{cr}}$:

(4) $$x_{\mathrm{cryst},\mathrm{macro}}=\frac{\rho -\rho_{\mathrm{am}}}{\rho_{\mathrm{cr}}-\rho_{\mathrm{am}}}$$

We therefore need to calculate $\rho_{\mathrm{am}}$ and $\rho_{\mathrm{cr}}$. While $\rho_{\mathrm{am}}$ is known from the equilibrated and quenched amorphous systems directly, we additionally need to evaluate a pure crystalline system. We setup and equilibrate a corresponding system as follows: We construct one chain as a straight line under consideration of the equilibrium bond length and bond angle. Then we place copies of this chain into the simulation box in an orthorhombic grid. This structure is preferred as it is known from experiments to be found in polyethylene under tensile loading [43]. Grid spacing is chosen to match the minima of the Lennard–Jones potentials as close as possible. The equilibration procedure is straightforward: We use Berendsen thermostat ($\tau_{\mathrm{thermo}}=1\;\mathrm{ps}$) and barostat ($\tau_{\mathrm{baro}}=1000\;\mathrm{ps}$) for quickly (100 ps) heating up the system to 233.15 K at dt=4fs. Subsequently we perform a relaxation of 5 ns followed by a production run of 5 ns. Other temperatures (293.15 K and 353.15 K) are reproduced by heating the equilibrated system at 233.15 K by a rate of 0.2 K/ps followed by further relaxation and production runs for 5 ns each. From the results of the production runs we sample the crystalline densities.

We determine a crystalline density of $0.935\pm 0.001\;{g/\mathrm{cm}}^{3}$ at 293.15 K. With respect to the coarse-grained model as well as our application, this result is in acceptable agreement with the experimental value of 0.99 g/cm^3^ [30]. The ratio of amorphous and crystalline densities is also in agreement with experimental results: We calculate for our simulation $\rho_{\mathrm{cr},\mathrm{sim}}/\rho_{\mathrm{am},\mathrm{sim}}=0.935/0.853\approx 1.096$ and for the experimental result $\rho_{\mathrm{cr},\exp}/\rho_{\mathrm{am},\exp}=0.99/0.87\approx 1.14$ [30]. This minor difference allows for a reliable determination of the degree of crystallinity in our polyethylene systems and ensures good comparability with experimental results. Remarkably, our result for the crystalline density is very close to the simulation results in [24]. On the basis of a comparable CG model by the use of analytic potentials they determine $\rho_{\mathrm{cr}}=0.93\;{g/\mathrm{cm}}^{3}$.

## 3. Results

In the following subsections, we describe the different effects that occur in priorly stretched polyethylene systems. We start by describing different system behavior depending on free and fixed boundary conditions. We here focus on short time frames of 40 ns. That span of time is sufficient to cover the initially distinct reactions of the systems. Furthermore, 40 ns are adequate to estimate how the systems evolve on a longer time scale as individual trends are already obvious from these results. Subsequently, we discuss temperature dependent and crystal growth effects. Finally, we present results from long simulation runs (up to 600 ns) by the example of two selected systems.

Our explanations and therefore all following figures start at the point when the systems are fully stretched to λ=6. From that point on, the systems are quenched from 500 K to 233.15 K, 293.15 K or 353.15 K, respectively, within 10 ns. Simultaneously, the systems are allowed to relax according to either free or fixed boundary conditions as described in Section 2.2.

### 3.1. Relaxation in Short Simulation Runs (40 ns)

#### 3.1.1. Chain Length Effects under Free Conditions

**Figure 1:**
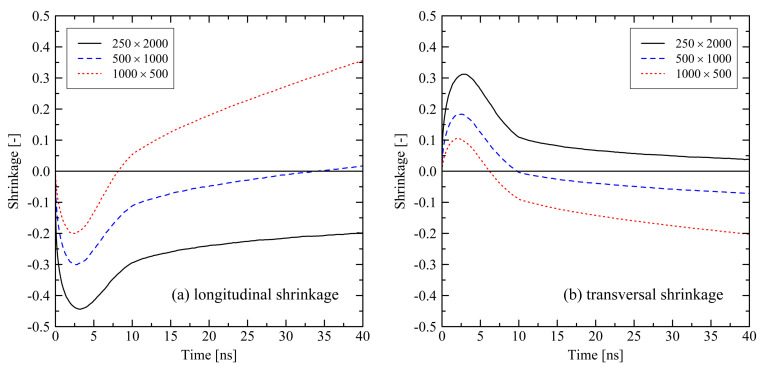
Longitudinal (**a**) and transversal shrinkage (**b**) for different system sizes M×N at target temperature of 293.15 K. The systems were simulated under free conditions. Each of the curves consists of 241 data points.

For the evaluation of relaxation effects in stretched polyethylene systems we first investigate the global shrinkage behavior. We define the longitudinal shrinkage as the relative change of the box dimensions in former stretching direction. Analogously, the transversal shrinkage is calculated as the average shrinkage in the lateral box dimensions. It is expected to observe longitudinal shrinkage in former stretching direction when the system undergoes conformational relaxation. Accordingly, in transversal direction we expect some swelling of systems. Figure 1 shows that expectations hold for the first approx. 3 ns after releasing the tensile stress. Subsequently, we observe swelling of the systems in longitudinal direction and shrinkage in transversal direction, which is initially unexpected. For shorter chains we notice that they show a more pronounced swelling (longitudinal direction) and shrinking (transversal direction) behavior past 3 ns after release.

**Figure 2:**
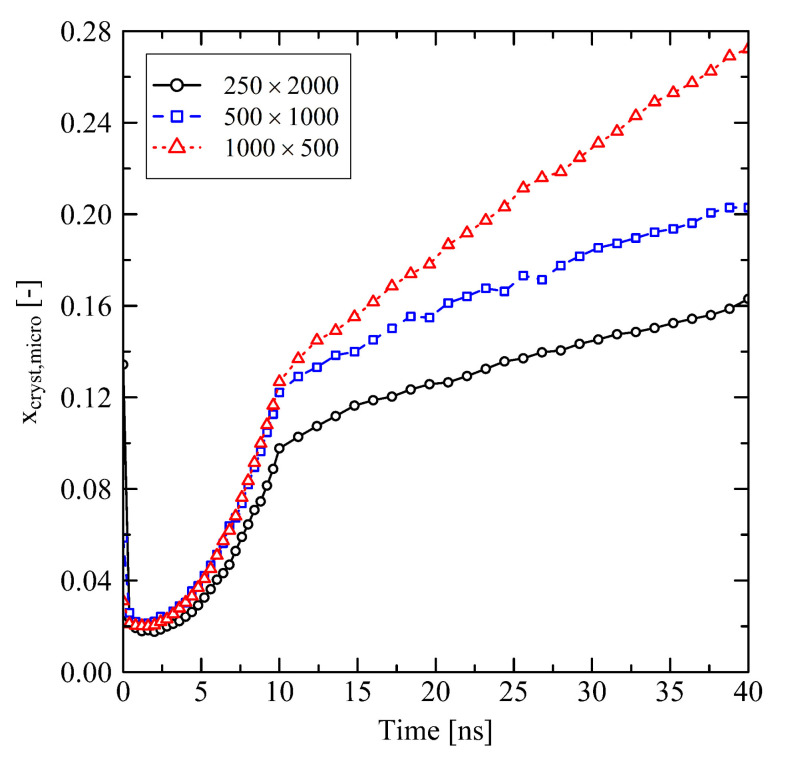
Degree of crystallinity based on the microscopic criterion ($x_{\mathrm{cryst},\mathrm{micro}}$) at different system sizes M×N. The systems were simulated under free conditions at target temperature of 293.15 K.

Investigating the micro-structure reveals that the orientation and crystallization effects drive the behavior during the relaxation of the systems. Evaluating the degree of crystallinity by the use of our micro-structural criterion, we clearly see that crystallization takes place. After about 3 ns, which corresponds to the starting of the longitudinal swelling (cf. Figure 1a), $x_{\mathrm{cryst},\mathrm{micro}}$ increases (Figure 2). From Figure 2 it is also clear that shorter chains crystallize faster than longer chains, especially in case of the N=500 system.

**Figure 3:**
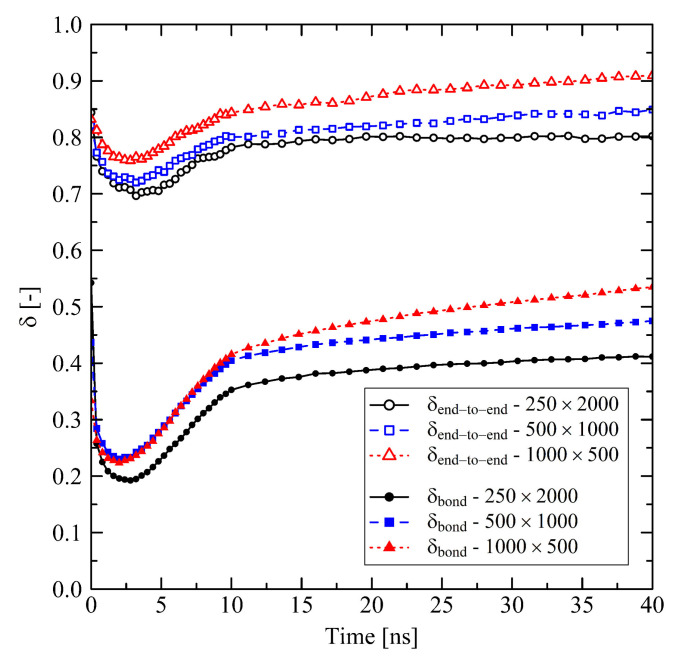
Orientation factor δ evaluated for single bonds and for the chain end-to-end vector for different system sizes M×N. The target temperature was set to 293.15 K. The systems were simulated under free conditions. A value of δ=1 represents full orientation in former stretching direction, wheras δ=0 describes purely amorphous orientation behavior.

Taking into account the orientation behavior of chain segments (bond vectors) and of chain end-to-end vectors (Figure 3), it becomes clear that after releasing the system a specific amount of orientation due to the former stretching is preserved, especially for the end-to-end vectors. Because of this globally pre-oriented state a dense crystalline packing takes predominantly place in the transversal direction. This leads to the observed transversal shrinkage behavior at t>3ns and also explains the subsequent swelling in longitudinal direction: Chain segments are forming into crystalline stems with parallel ordering in longitudinal direction. Consequently, local and global orientation of chains and chain segments increase in former stretching direction as it is seen from Figure 3.

**Figure 4:**
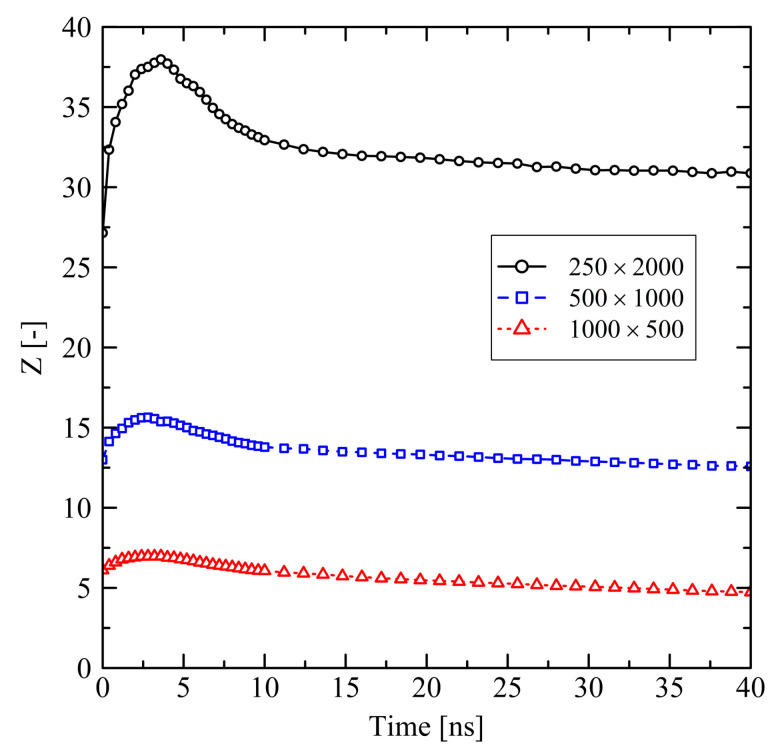
Average number of entanglements *Z* per chain for different system sizes M×N at target temperature of 293.15 K. The systems were simulated under free conditions.

Short chains with N=1000 and even more with N=500 show a remarkably flexible behavior as it is seen from Figure 1 (strong swelling after 3 ns) and Figure 2 (rapidly increasing level of crystallization). From theory this is expected, as shorter chains are generally more movable than longer chains as, for example, characterized by the disentanglement time $\tau_{d}∼N^{3.4}/N_{e}$, where *N* is the degree of polymerization and $N_{e}$ is the entanglement length. From this general relation it is clear that entanglement related effects play an important role. In our case, by analysing the PP we evaluate that the number of entanglements *Z* per chain at the end of the stretching (Figure 4, t=0ns) depends on the chain length. Furthermore, after releasing the systems, we observe that chains are re-entangling to an increasing extend with increasing chain length. For long chains (N=2000) these newly built entanglements are preserved to an higher extend, whereas short chains (N=500) evolve towards an entanglement level below their initial state. We conclude that the high number of entanglements *Z* in the long chain system is one crucial factor that prevents this system from rapid crystallization (Figure 2) and thus strong elongation (Figure 1).

#### 3.1.2. Chain Length Effects under Fixed Conditions

**Figure 5:**
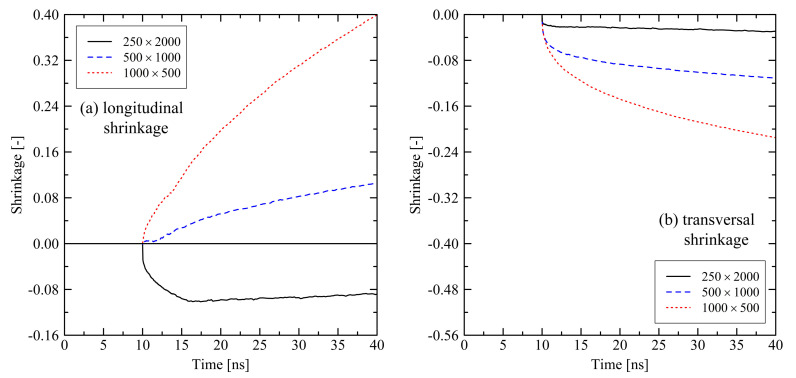
Longitudinal (**a**) and transversal shrinkage (**b**) for different system sizes M×N at target temperature of 293.15 K. The systems were simulated under fixed conditions. Each of the curves consists of 236 data points.

For fixed conditions, we observe a behavior that is comparable to the results from Section 3.1.1 only for the longest chains (N=2000). After cooling and releasing of the fixed box dimensions at 10 ns the longitudinal shrinkage is followed by a slight swelling (Figure 5a, system: 250×2000). In contrast systems with short chains (N≤1000) start to swell immediately after release, which was not observed for the corresponding investigations under free conditions. The previously observed swelling behavior in the transversal direction (cf. Figure 1b) is also now suppressed for all chain lengths.

**Figure 6:**
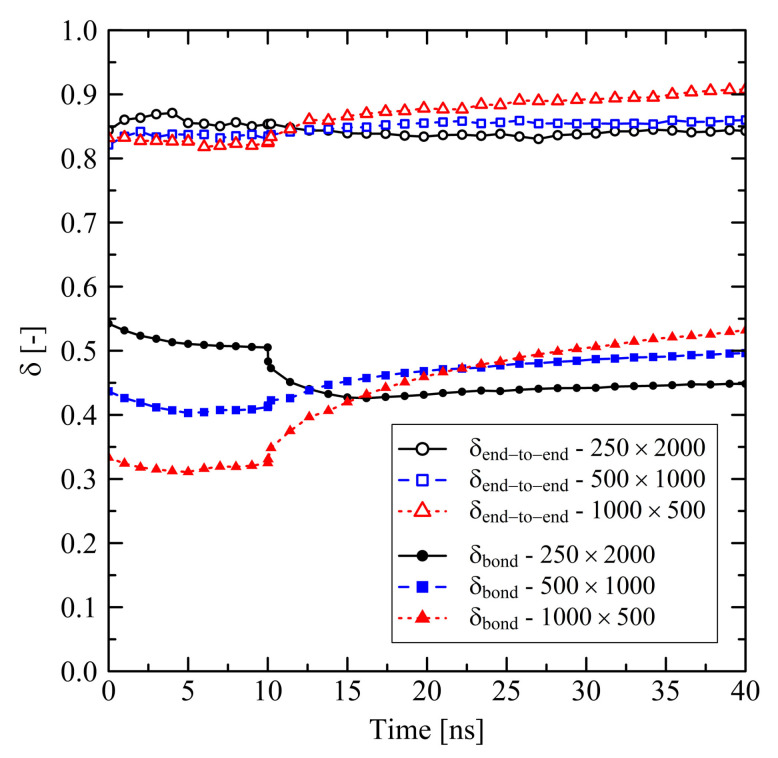
Orientation factor δ evaluated for single bonds and for the chain end-to-end vector for different system sizes M×N. The target temperature was set to 293.15 K. The systems were simulated under fixed conditions.

**Figure 7:**
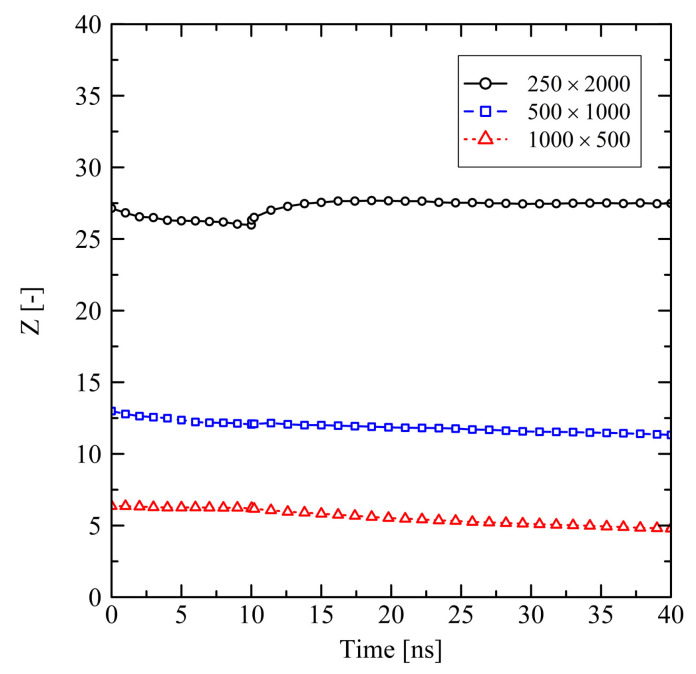
Average number of entanglements *Z* per chain for different system sizes M×N at target temperature of 293.15 K. The systems were simulated under fixed conditions.

Investigations of the effects on the microscale reveal that the level of orientation of bond vectors after stretching (t=0ns) depends on the chain length (Figure 6). For longer chains we observe a higher level of bond vector orientations. We explain this behavior by the higher number of entanglements *Z* per chain for longer chains (Figure 7): The single entanglements act as virtually fixed points between which the orientation of local bonds is built up during stretching. Throughout the subsequent 10 ns holding and cooling stage bond orientations are only weakly relaxing (Figure 6, 0≤t≤10 ns) as the system size is globally fixed. The level of the chain end-to-end vector orientations remains at a constant and remarkably high level (Figure 6).

**Figure 8:**
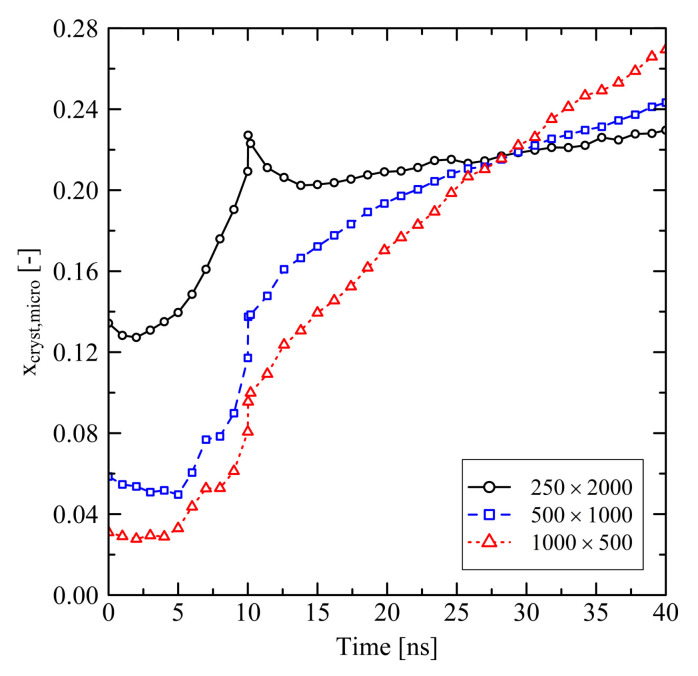
Degree of crystallinity based on the microscopic criterion ($x_{\mathrm{cryst},\mathrm{micro}}$) at different system sizes M×N. The systems were simulated under fixed conditions at target temperature of 293.15 K.

After releasing the systems at 10 ns, local structures in the long chain system (N=2000) change significantly. This is indicated by the spontaneous decrease of orientation of local bond vectors (Figure 6) as well as the mild increase of the number of entanglements *Z* per chain (Figure 7). The long chains are re-entangling up to a certain level. At the moment of release the elastic conformational energy that was previously saved between the entanglement points is now instantaneously released. This leads to a disturbance in the long chain system, which also causes a small initial dissolution of crystalline zones after release (Figure 8). That effect is followed by a slow increase of crystallinity. Systems with short chains (N≤1000) develop in a different way: The generally low level of entanglements per chain (Figure 7) in combination with strong global orientation (Figure 6) gives the chains the ability to easily pass along each other in longitudinal direction. This leads to the observed strong swelling in that specific direction (Figure 5). In transversal direction these systems severely shrink. All these observations correlate with a rapid increase of local orientations (Figure 6) as well as level of crystallinity (Figure 8).

### 3.2. Temperature Dependent Effects and Crystal Growth

**Figure 9:**
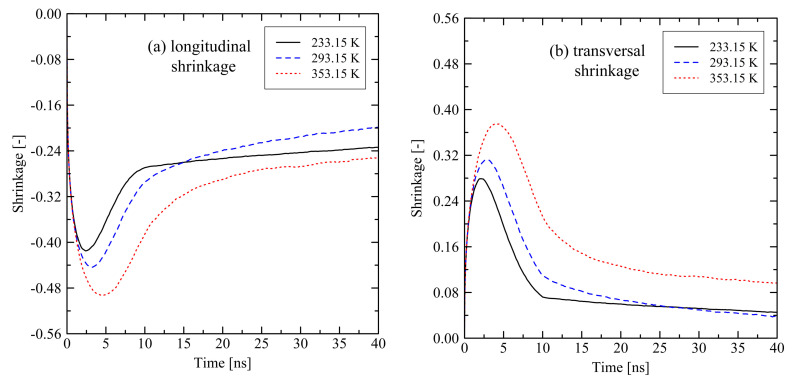
Longitudinal (**a**) and transversal shrinkage (**b**) for a 250×2000 (M×N) system at different temperatures. The systems were simulated under free conditions at target temperature of 293.15 K. Each of the curves consists of 241 data points.

With respect to temperature dependent effects on the relaxation of stretched polymers Figure 9 gives insight into the shrinking and swelling behavior. Here, we only present results for investigations under free conditions. The general trend does not differ between the investigated temperatures. A longitudinal shrinkage is followed by a swelling, whereas in the transversal direction, we observe swelling followed by shrinkage over time.

**Figure 10:**
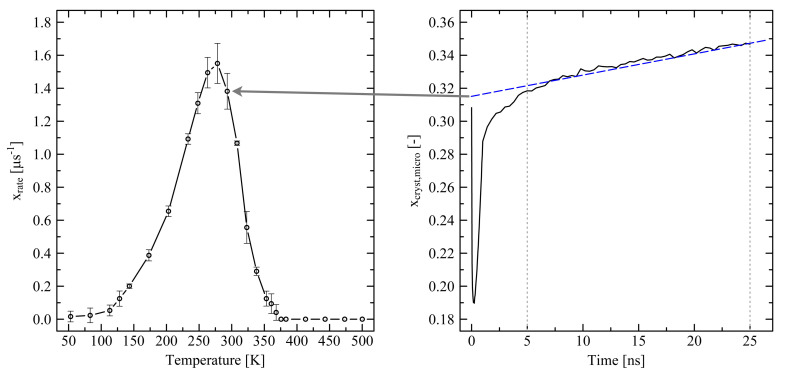
Temperature dependent crystallization rate ($x_{\mathrm{rate}}$) determined for a 250×2000 (M×N) system under free conditions (left). System was quenched from 500 K to the specific target temperatures within 1 ns. The graph on the right shows the determination of the crystallization rate by the example at 293.15 K: We define the crystallization rate from the average slope of the increasing degree of crystallinity according to our microscopic definition between 5 and 25 ns (blue dashed line). As we here take the growth of initially very small crystallites into account, we set $n_{\mathrm{stem}}\geq 1$ (cf. Section 2.3). Lines in the figure on the left side, as well as the dotted lines in the right figure, are a guide to the eye only. For more clarity, note that scales in the figures are slightly shifted inwards. The figure on the right side consists of 86 data points.

Interestingly, the simulation at 293 K shows exceptional behavior as these results do not fully fall in line with results at the other investigated temperatures (see crossing of curves in Figure 9 at approx. 15 ns (longitudinal direction) and 27 ns (transversal direction)). The shrinkage/swelling effects at 293 K are stronger in comparison to the results at other temperatures. We observe the same trend for the systems under fixed conditions. Figure 10 reveals that the temperature dependent crystallization rate dominates the observed behavior. From the figure it is clear that the stronger swelling of the system at 293 K is due to a quicker growth of crystalline zones, which is 1.3 times (11.1 times) faster than at 233 K (353 K).

Figure 10 also provides an insight into the temperature range where crystallization is thermodynamically possible. From 375.65 K on our models show that no crystal growth takes place ($x_{\mathrm{rate}}=0$). Above that specific temperature small initial crystallites are immediately destroyed as the level of kinetic energy of the chains is too high. Experimental results from DSC (differential scanning calorimety) in [44] reveal that depending on the cooling rate the onset crystallization temperature $T_{c,on}$ for high-density polyethylene varies from 393.75K (cooling rate 2.5K/min) to 389.65K (20K/min). By using fast DSC Toda et al. [45] report $T_{c,on}\approx 379\;K$ (cooling rate 1200K/s). Considering the very fast cooling rates in the simulations on the microscale our result is in good agreement with experiments. At very low temperatures we observe that the crystallization rate does not strictly fall to zero. Due to the quick cooling of the systems densification towards a final equilibrium state is still in progress. Hence, very few crystallites are able to form, despite the very low temperature. Additionally, beads are denser packed at low temperatures. As our microscopic crystallinity criterion uses a constant threshold distance to differentiate between crystalline and amorphous states of neighboring beads, our evaluation method has a very slight tendency to overestimate the degree of crystallinity at very low temperatures. Therefore, our simulation results are showing the observed crystallization rates at very low temperatures.

**Figure 11:**
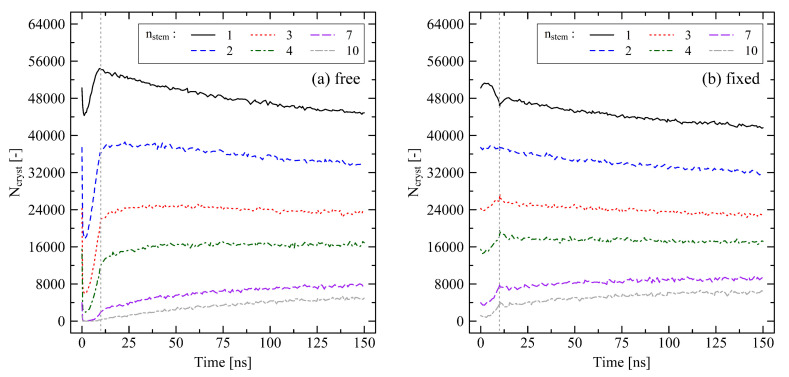
Number of beads in crystalline state ($N_{\mathrm{cryst}}$) depending on the crystal stem length determined for a 250×2000 (M×N) system. We here use our microscopic crystallinity criterion (cf. Section 2.3) for the identification of the number of beads that belong to a specific stem length ($n_{\mathrm{stem}}=\;1,\;2,\;3,\;4,\;7,\;10$). (**a**) Results for free conditions. (**b**) Results for fixed conditions. The target temperature was set to 293.15 K. The grey dotted lines indicate the end of the cooling at 10 ns. For clear visibility of initial effects, note that scales in the figures are slightly shifted inwards. Each of the curves consists of 160 data points.

For a deeper insight into crystallization kinetics we investigate crystal growth. We therefore use the microscopic criterion for the observation of beads that are in a crystalline state. We here count the number of beads that belong to one specific crystal stem length according to our criterion. By evaluating various crystal stem lengths $n_{\mathrm{stem}}$ between one and ten beads we are able to monitor the growth of crystallites in the systems. In Figure 11 we compare the different crystallization behavior under free and fixed conditions. Results are sampled over 150 ns (cf. Section 3.3).

Under free conditions (Figure 11a), the number of crystallites, which have previously been formed due to the stretching of the system, initially drops to a significant degree. An instantaneous release of the system severely disturbs the early ordering of crystalline regions. The temperature at the very first moment of release is still at 500 K so that dissolution of crystallites is likely. The generally large number of single crystalline beads indicates that there is a great amount of crystal nuclei in the stretched system. During cooling and densification of the system we observe an increasing tendency for growing crystals especially for mid-sized crystals ($n_{\mathrm{stem}}=$ 2, 3, 4). After the cooling stage is finished (t=10ns), crystal growth on the larger sized level ($n_{\mathrm{stem}}\geq 7$) is monitored. In contrast, the number of nuclei ($n_{\mathrm{stem}}=1$) is significantly decreasing as they are either unstable or grow to larger crystallites.

In comparison, the development of crystallites under fixed conditions (Figure 11b) is considerably different in the beginning. Holding the box dimensions fixed during the cooling stage leads to a growth of crystalline structures on the mid and long investigated scales ($n_{\mathrm{stem}}\geq 3$). The number of nuclei ($n_{\mathrm{stem}}=1$) decreases during the cooling and holding stage as many of these are already transformed to larger crystallites. At the moment of release of the system at t=10ns the internal structure of the system is only very slightly disturbed by its sudden ability to change its size. Hereinafter, a trend, as seen for the for free conditions, towards slowly growing large crystallites ($n_{\mathrm{stem}}\geq 7$) is obvious.

### 3.3. Relaxation in Long Simulation Runs (150 and 600 ns)

As for all investigated systems we do not observe a final equilibrium state, where especially the degree of crystallinity reaches a stable plateau, we perform longer simulation runs for two particular system sizes. We simulate one large system with 250 chains at chain length 2000 for 150 ns and one small system with 100 chains at chain length 1000 for 600 ns at 293.15 K. As the general development of the systems on the long time scale does not strongly depend on the boundary conditions, we here only concentrate on the results of the simulations under fixed box dimensions during cooling (cf. Section 3.1.2).

**Figure 12:**
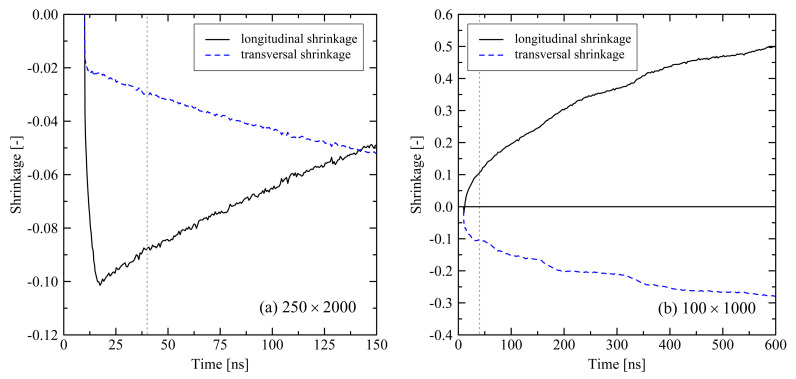
Evolution of the longitudinal and transversal shrinkage on an extended time scale (150 and 600 ns) for different system sizes M×N (250×2000 (**a**), 100×1000 (**b**)). The target temperature was set to 293.15 K. The systems were simulated under fixed conditions. The grey dotted lines mark t=40ns. Each of the curves consists of 256 data points.

From Figure 12 it is clearly visible that, even after long simulation runs, a final system state is not recorded. Still, simulations show swelling in longitudinal direction, but a trend towards a plateau regime is visible for the 600 ns run.

**Figure 13:**
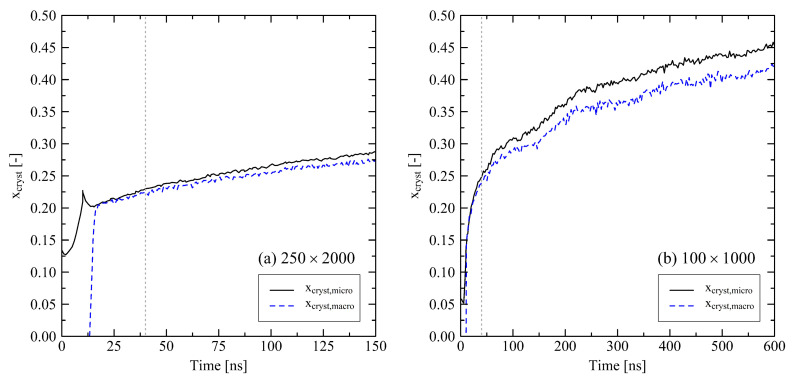
Degree of crystallinity based on the microscopic criterion ($x_{\mathrm{cryst},\mathrm{micro}}$) and the ratio of crystalline and amorphous densities ($x_{\mathrm{cryst},\mathrm{macro}}$) for a 250×2000 (**a**) and a 100×1000 system (**b**). The systems were simulated under fixed conditions by cooling from 500 K to 293.15 K within 10 ns. As we defined the results for $x_{\mathrm{cryst},\mathrm{macro}}$ on the basis of densities at 293.15 K, the results for the macroscopic degree of crystallinity up to t=10 ns are not comparable to the microscopic definition. The grey dotted lines mark t=40ns. Each of the curves consists of 256 data points. Only the curve representing the 250×2000 system and $x_{\mathrm{cryst},\mathrm{micro}}$ definition consists of 160 data points.

**Figure 14:**
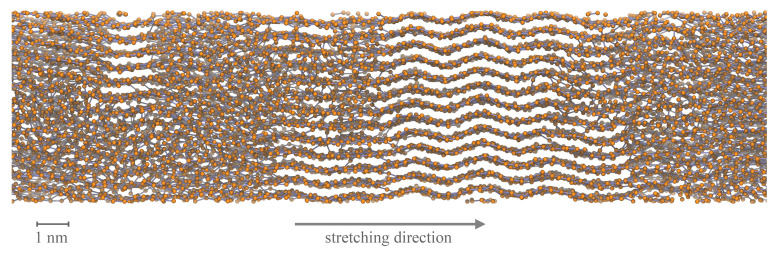
Cutout from a 100×1000 (M×N) system after 600 ns simulation. The system was simulated under fixed conditions at target temperature 293.15 K. The semi-crystalline structure is visible. Additionally, the distinctive orientation of chain segments within crystalline regions in prior stretching direction is obvious.

As already discussed, crystallization effects are driving the system behavior. Figure 13 shows that also on a longer time scale longitudinal stretching of the systems is continuously connected to an increasing degree of crystallinity. By evaluating the degree of crystallinity a stable state for the 100×1000 system is expected to be close. A meta-stable state at around 500 ns already appears in Figure 13b. A cutout from a 100×1000 system with clear semi-crystalline structure at the end of the simulation (600 ns) is shown in Figure 14. Beyond that, by plotting the degree of crystallinity according to the microscopic and macroscopic definition we see that both criteria develop in a very similar manner. There is only a slight offset between the two definitions. Hence, we conclude that the microscopic definition (with $n_{\mathrm{stem}}\geq 3$) is suitable for the evaluation of the degree of crystallinity.

**Figure 15:**
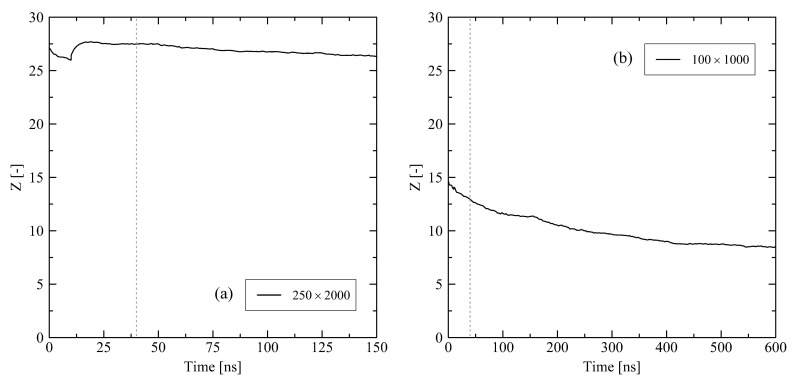
Average number of entanglements *Z* per chain for a 250×2000 (**a**) and a 100×1000 system (**b**) (M×N). The systems were simulated under fixed conditions at target temperature of 293.15 K. The grey dotted lines mark t=40ns. The curve representing the 100×1000 system consists of 256 data points. The curve representing the 250×2000 system consists of 160 data points.

Evaluating the number of entanglements (Figure 15), tendencies observed in short simulations continue. While the mid-sized chains significantly disentangle (Figure 15b, 100×1000: Z(0ns)=14.7, Z(150ns)=11.4, Z(600ns)=8.5), the long chain system remains in a significantly more entangled state (Figure 15a, 250×2000: Z(0ns)=27.2, Z(150ns)=26.3). Again, after 600 ns, a final state is not fully captured within the simulated time frame, but is close.

## 4. Discussion

By investigating stretched and subsequently cooled polyethylene systems under different loading conditions, we see remarkable effects on the microscale. If the system is in a temperature range where crystallization is thermodynamically possible (cf. Figure 10), crystallization effects strongly dominate the behavior after release of the tensile strain. Further visco-elastic relaxation effects play only a secondary role as we detect longitudinal elongation in all systems when crystallization sets in. Longitudinal elongation is also reported and explained by chain straightening and alignment during crystallization in [20].

Comparing the evolution of systems under free and fixed conditions, we initially monitor distinctively different behavior concerning orientation, crystallization and shrinkage. On the longer time scale (t≫10ns) these differences become less prominent. However, it is shown that the degree of crystallinity in systems, which were processed by the use of fixed boundary conditions, is significantly higher at the end of the simulations (t=40ns), at least for chain lengths N≥1000 (cf. Figure 2 and Figure 8). Regarding the investigated system sizes, results reveal that systems with shorter chain length (N≤1000) tend to strongly elongate in longitudinal direction (cf. Figure 5 and Figure 12). Very short chains (N=500) especially have a tendency to almost completely disentangle, which is unlikely for a real polymer that is used in industrial application. Only by the use of long chains (N=2000) we are able to reproduce a system behavior which is relevant on the macroscopic scale. This is in contrast to our results from tensile tests [27], where the behavior of a system with chain length N=500 was already comparable with systems with larger chain lengths and real-life polymers, respectively. This emphasizes that chain length has a strongly varying influence on different physical quantities. Additionally, due to limited resources we are not able to fully cover the physical time scale that is needed for a complete reproduction of relaxation and crystallization effects. Nevertheless simulations over 600 ns show a clear way towards an equilibrium state (Figure 12).

Finally, our model is able to reproduce the crystallization onset temperature very close to the experimental results (Figure 10). Comparing the overall trend of the crystallization rate our results differ from experiments more significantly. Especially the peak crystallization rate ($T_{\mathrm{peak}}=278.15\;K$, $x_{\mathrm{rate},\mathrm{peak}}=1.60\pm 0.10\;\mu$s${}^{-1}$) is shifted towards lower temperatures compared to experiments showing that the peak crystallization rate is in the range between 343.15 K and 348.15 K [46]. Using MD simulation on the basis of an UA approach Yamamoto [47] determines the peak crystallization temperature at around 330 K.

Our models are based on monodisperse polyethylene chains. It needs to be proved how polydisperse systems affect results. The current model is also limited to the investigated time and length scale. From our results we see a clear influence of the chain length on the results. Analysis of longer chains (N>2000) on a longer time scale is needful for covering a range closer to the macroscopic scale.

## 5. Summary

In this study, we analysed the relaxation and crystallization behavior of stretched and subsequently cooled polyethylene systems for up to 600 ns. We used two different approaches: (a) “free” conditions, which allow the systems to contract instantaneously after stretching and (b) “fixed” conditions, which hold the box dimensions fixed during solidification of the melt. Only after cooling to specific temperatures these systems are allowed to change its shape and size. Both procedures represent loading conditions that occur in real-life polymer processing.

By using large systems with chain lengths of 2000 beads, we are able to realistically model entanglement, crystallization and relaxation behavior of polyethylene. Our results clearly show, that cooling under fixed and free conditions, respectively, leads to substantially different crystallization kinetics on the microscale: The use of free conditions results in a strong dissolution of orientations and initially generated crystallization nuclei. Re-entanglement effects, evaluated by the primitive path analysis, play an important role, especially in case of large chain lengths (N=2000). In contrast, for fixed conditions, we determine significant conservation of orientations and crystalline nuclei during relaxation. In this case re-entanglement effects have a minor influence on the micro-structure.

Chain length has an essential effect on the results. Only a chain length of 2000 gives results that are comparable with polymer behavior on the macroscale. Shorter chains show a trend to almost completely disentangle after strong elongation. From our results we are able to determine the temperature dependent rate of crystallization. The crystallization onset temperature is reproduced in good agreement with experimental results. The course of the crystallization rate is also comparable to experimental results.

Depending on real-life process conditions, in many cases, not only does uniaxial stretching occur. The blow-molding and deep drawing processes especially induce biaxial stretch ratios during part formation. Therefore, investigations of the influence of biaxial stretching on the microscopic structure is needed. In a future study, we will address both the microscopic structure due to biaxial stretching and resulting relaxation effects. 

## Figures and Tables

**Table 1:** Bonded and Lennard–Jones force-field parameters for coarse-grained polyethylene [26]. Note that the Lennard–Jones parameters depend on the particle position.

Bead Type	Bond Length		Bond Angle		Dihedral Angle	
	$b_{0}$	$K_{b}$	$\theta_{0}$	$K_{\theta }$	m	$K_{\phi }$
	**[nm]**	**[kJ mol${}^{-1}$ nm${}^{-4}$]**	**[${}^{\circ }$]**	**[kJ mol${}^{-1}$]**	**[-]**	**[kJ mol${}^{-1}$]**
CG3	0.353	19730	146.4	56.6	1	0.74
**Bead Type**	**Position**	σ	ϵ	$\sigma_{1\text{-}4}$	$\epsilon_{1\text{-}4}$	
		**[nm]**	**[kJ mol${}^{-1}$]**	**[nm]**	**[kJ mol${}^{-1}$]**	
CG3mid	middle	0.457	2.214	0.401	2.213	
CG3end	end	0.468	2.415	0.421	2.415	

**Table 2:** Comparison of different physical properties of polyethylene determined by use of the CG force field from [26] (system size: 250×2000 (M×N)) and experimental results. Unless otherwise noted, values are determined in this work. For the experimental value of the amorphous density at 293 K the corresponding author does not provide standard errors.

	$\rho_{\mathrm{amorph},293K}$	$\rho_{493K}$	CTE	*T*g
	[g cm${}^{-3}$]	[g cm${}^{-3}$]	[$1\times 10^{-4}$ K${}^{-1}$]	[*K*]
sim.	0.853±0.001	0.749±0.001	^1^2.015±0.013 [27]	210±3 [27]
exp.	0.87 [30]	0.747±0.002 [31]	^2^0.95±0.05 [32]	195±10 [33]
			^3^2.19±0.03 [32]	

^1^ determined by cooling from 333.15 K to 273.15 K; ^2^ injection molded sample, temperature range 298.15 K to 328.15 K, perpendicular to injection direction; ^3^ injection molded sample, temperature range 333.15 K to 373.15 K, in injection direction.

## Data Availability

The data that support the findings of this study are available from the corresponding author upon reasonable request.
